# Maternal serum NGAL in the first trimester of pregnancy is a potential biomarker for the prediction of gestational diabetes mellitus

**DOI:** 10.3389/fendo.2022.977254

**Published:** 2022-11-16

**Authors:** Ling Lu, Chanyu Li, Jie Deng, Jianbo Luo, Chaolin Huang

**Affiliations:** Department of Gynaecology, The First Affiliated Hospital of Chengdu Medical College, Chengdu, Sichuan, China

**Keywords:** biomarker, first trimester, gestational diabetes mellitus, neutrophil gelatinase-associated lipocalin (NGAL), insulin resistance

## Abstract

**Objective:**

Gestational diabetes mellitus (GDM) has adverse effects on the health of mothers and their offspring. Currently, no known biomarker has been proven to have sufficient validity for the prediction of GDM in the first trimester of pregnancy. The aim of this study was to investigate the potential relationship between serum neutrophil gelatinase-associated lipocalin (NGAL) levels in the first trimester of pregnancy and later GDM risk and to evaluate the performance of serum NGAL as a biomarker for the prediction of GDM.

**Methods:**

The study was conducted by recruiting participants at 8–13 weeks of gestation from The First Affiliated Hospital of Chengdu Medical College between January and June 2021; participants were followed up for oral glucose tolerance test (OGTT) screening at 24–28 gestational weeks. We examined the serum NGAL levels of all subjects in the first trimester who met the inclusion and exclusion criteria. Anthropometric, clinical, and laboratory parameters of the study subjects were obtained during the same study period. A logistic regression model was carried out to investigate the potential relationship between serum NGAL levels in the first trimester of pregnancy and later GDM risk. The receiver operating characteristic (ROC) curve and area under the curve (AUC) were used to assess the discrimination and calibration of serum NGAL as a biomarker for the prediction of GDM in the first trimester of pregnancy.

**Results:**

Serum NGAL levels in the first trimester of pregnancy were significantly higher in women who later developed GDM than in those who did not develop GDM. Serum NGAL levels in the first trimester of pregnancy were positively associated with an increased risk of GDM after adjustment for potential confounding factors. The risk prediction model for GDM constructed by using serum NGAL levels in the first trimester of pregnancy achieved excellent performance.

**Conclusions:**

Maternal serum NGAL in the first trimester of pregnancy is a potential biomarker for the prediction of GDM, which could help guide the clinical practice of antenatal care.

## Introduction

Gestational diabetes mellitus (GDM) is a common gestational disorder characterized by glucose intolerance during the second or third trimester (1, 2). Depending on ethnicity and the screening methods and diagnostic tests used, it is estimated that GDM occurs in 6-18% of all pregnancies worldwide (3–5). GDM affects approximately 15% of all Chinese pregnant women according to the International Association of Diabetes diagnostic criteria for GDM (6).

Currently, GDM is diagnosed with an oral glucose tolerance test (OGTT), which is generally performed in the second trimester of pregnancy. However, according to the recent guidelines of the American Congress of Obstetricians and Gynecologists (ACOG), early screening in the first trimester is recommended in women with risk factors for GDM (e.g., BMI above 25, hypertension, family history of diabetes, and known impaired glucose metabolism) (7). Moreover, the effective early detection of women at risk in the first trimester could be beneficial for reducing disease onset and associated maternal and perinatal complications by providing timely interventions such as physical exercise and dietary changes (8). Therefore, it is necessary to identify some appropriate biomarkers that would allow early prediction of GDM risk.

Neutrophil gelatinase-associated lipocalin (NGAL), also known as lipocalin-2 (LCN-2), is a 25-kDa secretory glycoprotein that was originally identified in mouse kidney cells and human neutrophil granules (9–11). It plays an important role in the regulation of the immune response, inflammation, and tumour metastasis (9, 12–14). In addition, several studies have also reported that NGAL is associated with obesity through the induction of IFNγ expression, resulting in subsequent adipogenesis in adipose tissues (15–17). In addition to the close associations of NGAL with obesity observed in previous studies, it has also been reported that NGAL plays an important role in the pathophysiology of other metabolic diseases, such as dyslipidaemia, dysglycaemia, and bone metabolic disease (18–20). However, the role of NGAL in regulating blood glucose levels is controversial, as different studies have reported discrepant results. Although some studies suggest that NGAL has a role in promoting glucose intolerance, insulin resistance, and obesity, there is also evidence related to its beneficial anti-diabetic role (16, 19, 21). Several studies reported that circulatory NGAL levels were elevated and positively correlated with obesity, hypertriglyceridaemia, hyperglycaemia, and insulin resistance in type 2 diabetes mellitus patients (16, 22). In contrast, other studies reported that NGAL has an important role in improving insulin sensitivity and glucose metabolism, either by stimulating insulin secretion or by controlling the food intake behaviours of mice (19, 23). Therefore, the above findings suggest that NGAL plays an important role in glucose homeostasis, making it a potential new biomarker of abnormal glucose metabolism during pregnancy.

To the best of our knowledge, maternal serum NGAL levels in the first trimester of pregnancy in women with GDM have been little studied and are poorly understood. Two previous studies reported that maternal serum NGAL levels were significantly higher in women with GDM than in those without GDM and correlated positively with fasting plasma glucose in the third trimester (24, 25). However, the studies were conducted in the third trimester when OGTT screening had already been performed. Thus, it may not be helpful for the early detection of GDM in the first trimester of pregnancy, as the levels of NGAL may be different between the first trimester and third trimester. The aim of this study was to investigate the potential relationship between serum NGAL levels in the first trimester of pregnancy and later GDM risk and to evaluate the performance of serum NGAL as a biomarker for the prediction of GDM.

## Materials and methods

### Study participants and design

The prospective cohort study was conducted by recruiting participants at 8–13 weeks of gestation from The First Affiliated Hospital of Chengdu Medical College between January and June 2021; participants were followed up for OGTT screening at 24–28 gestational weeks. A total of 824 pregnant women visited the antenatal clinic at the hospital for their first prenatal examination during the study period. The inclusion criteria were as follows: (1) women with a singleton pregnancy; (2) women between 18 and 40 years of age; (3) women at 8–13 weeks of gestation; (4) women who planned to receive antenatal care and deliver in the study hospital; and (5) women who signed the consent form. The exclusion criteria were as follows: (1) women with a previous history of chronic diseases such as chronic kidney disease, diabetes, hypertension, polycystic ovary syndrome, malignant tumours, autoimmune diseases, blood system diseases, or infectious diseases; (2) women with foetal malformation or those who experienced miscarriage; and (3) women with incomplete or unavailable data. A total of 516 participants met the inclusion and exclusion criteria and had ELISA performed for serum NGAL detection at 8-13 gestational weeks. However, 28 participants were lost to follow-up because of miscarriage or opting to receive prenatal care at other hospitals. Ultimately, 488 women were recruited for the study, and 74 were diagnosed with GDM by OGTT screening at 24–28 gestational weeks. The overall sample size was calculated using the following formula as described in previous studies: n= Z^2^×P×(1-P)/e^2^, where n= the required sample size, Z= 1.96 at a 95% confidence interval (CI), P= the prevalence of GDM (14.8%) and e= the margin of error (5%) (6, 26, 27). The total sample size was calculated to be 194. However, 488 eligible participants were recruited for this study. PASS (Power Analysis and Sample Size) software was used to calculate the statistical power, effect size, and smallest sample size for different statistical tests.

**Figure 1:**
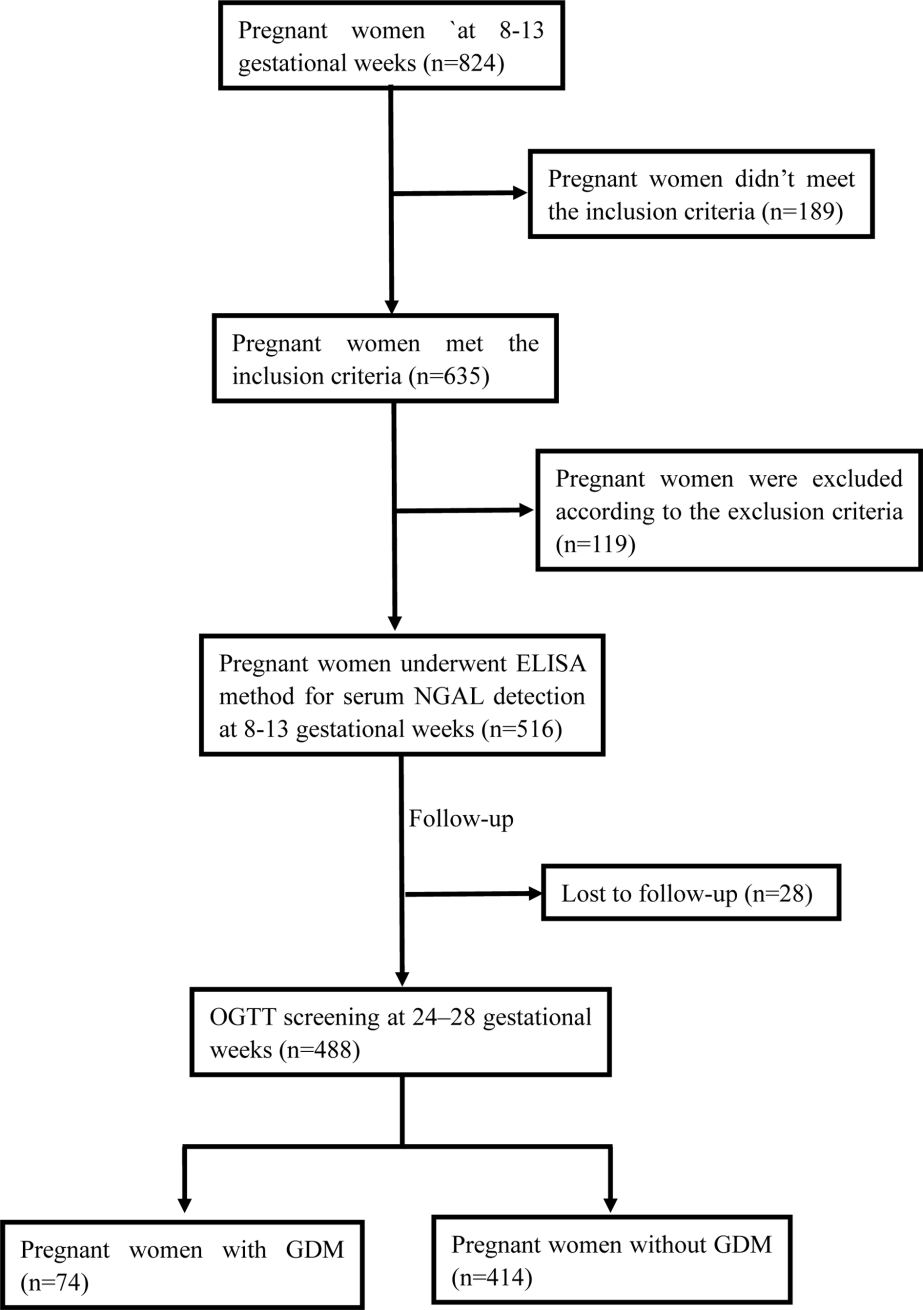
Flow graph of the study design.

The study flowchart is presented in **Figure 1**. The study was approved by the Ethics Committee of The First Affiliated Hospital of Chengdu Medical College (No. CYFY17032024). Informed consent was obtained from all participants, and all procedures were conducted according to the Declaration of Helsinki.

### Assessment of GDM and glucose homeostasis

A 75-g OGTT was performed to screen for GDM at 24–28 gestational weeks. According to the criteria of the American Diabetes Association, GDM was diagnosed when any of the following plasma glucose values were met or exceeded: a fasting glucose level ≥5.1 mmol/L, a 1-h glucose level ≥10.0 mmol/L, or a 2-h glucose level ≥8.5 mmol/L (1). The homeostasis model assessment of insulin resistance (HOMA-IR) was calculated according to published formulas (28).

### Clinical characteristics and laboratory tests

Basic information, including name, age, prepregnancy weight, height, gravidity and parity, diastolic blood pressure (DBP), systolic blood pressure (SBP), and history of diseases, was collected by trained nurses when the study subjects visited the obstetrics clinic for their first prenatal examination. For the laboratory tests, fasting plasma samples taken at 8–13 weeks of gestation were used. Blood glycosylated haemoglobin (HbA1c), serum insulin, uric acid, serum creatinine (Cr), high-density lipoprotein cholesterol (HDL-C), low-density lipoprotein cholesterol (LDL-C), triglyceride (TG), total cholesterol (TC), alanine aminotransferase (ALT), and aspartate transaminase (AST) levels were measured on the same day by standard methods in the clinical laboratory of our hospital.

The participants’ serum NGAL levels were detected by using a commercially available enzyme-linked immunosorbent assay kit (Abcam, Shanghai, China). All samples were analysed in three duplicates according to the manufacturer’s instructions.

### Statistical analysis

Data were analysed by using SPSS software (version 16.0, Chicago, IL, USA). Data with normal distributions are presented as the mean ± SD, and nonnormally distributed data are presented as frequencies. To compare differences between the GDM group and the non-GDM group, the independent-samples *t* test or Wilcoxon test was used for continuous variables, and the chi-square test or Fisher’s exact test was used for categorical variables. The relationship between serum NGAL levels and clinical and laboratory parameters was determined by Pearson correlation analysis. Logistic regression was used to analyse the association between GDM risk and serum NGAL levels with adjustment for potential confounding factors. The results are presented as the odds ratio (OR) and 95% CI. The receiver operating characteristic curve (ROC) and area under the curve (AUC) were used to evaluate the specificity and sensitivity of serum NGAL as a potential biomarker for the prediction of GDM. A *p <*0.05 was considered statistically significant.

## Results

### Clinical and laboratory characteristics of the study participants

The clinical and laboratory characteristics of the GDM group and non-GDM group are shown in **Table 1**. There were no statistically significant differences between the two groups in gestational age, parity, SBP, or DBP at sampling. However, maternal age and pre-pregnancy BMI were significantly higher in the GDM group than in the non-GDM group. Regarding laboratory characteristics, serum HbA1c, fasting insulin, TC, TG, LDL, OGTT glucose and HOMA-IR levels were significantly higher in the GDM group than in the non-GDM group (*P*<0.05). However, no differences in HDL-C, AST, ALT, serum creatinine, or uric acid levels were observed between the two groups (*P*>0.05). We also observed that the serum NGAL levels were significantly higher in the GDM group than in the non-GDM group (*P <*0.001, **Table 1**).

**Table 1:** Clinical and laboratory characteristics of the study participants.

Parameters	GDM group (n=74)	non-GDM group (n=414)	*p-value*
Maternal age (years)	28 ± 3.45	26 ± 3.23	**0.013**
Gestational age (weeks)	11.23 ± 2.43	11.75 ± 2.71	0.214
Pre-pregnancy BMI (kg/m^2^)	25.47 ± 4.67	23.46 ± 4.22	**0.023**
SBP (mm/Hg)	116.17 ± 12.32	114.78 ± 15.72	0.128
DBP (mm/Hg)	75.52 ± 9.34	72.48 ± 8.67	0.273
Parity			0.899
Primiparous	39	212	
Multiparous	35	202	
HbA1c (%)	5.51 ± 0.82	4.22 ± 0.78	**0.024**
Fasting insulin (mIU/ml)	9.73 ± 0.21	7.2 ± 0.13	**<0.001**
Fasting glucose (mmol/L)	5.45 ± 1.29	4.28 ± 0.63	**0.031**
1 h-glucose (mmol/L)	10.09 ± 1.91	7.35 ± 1.82	**0.027**
2 h-glucose (mmol/L)	8.31 ± 1.21	6.72 ± 1.29	**0.012**
HOMA-IR	2.18 ± 0.89	1.05 ± 0.22	**<0.001**
TC (mmol/L)	6.62 ± 0.61	5.12 ± 0.53	**0.021**
TG (mmol/L)	2.73 ± 0.21	1.62 ± 0.14	**0.022**
LDL-C (mmol/L)	4.65 ± 0.54	3.64 ± 0.47	**0.017**
HDL-C (mmol/L)	2.51 ± 0.13	2.09 ± 0.23	0.318
AST (U/L)	21 ± 3.27	22 ± 3.95	0.223
ALT (U/L)	19 ± 3.58	20 ± 3.14	0.274
Creatinine (μmol/L)	69 ± 6.73	71 ± 7.45	0.121
Uric acid (μmol/L)	305 ± 16.56	312 ± 18.27	0.217
Serum NGAL levels (ng/ml)	72.37 ± 8.24	47.25 ± 7.38	**<0.001**

Data are presented as the means ± standard deviations for continuous variables or frequencies for categorical variables. P values were calculated by using independent-samples t tests for normally distributed continuous variables and chi-square tests for categorical variables. Statistically significant values at P < 0.05 are shown in bold.

BMI, body mass index; SBP, systolic blood pressure; DBP, diastolic blood pressure; HbA1c, glycated haemoglobin; HOMA-IR, homeostasis model assessment-insulin resistance; TC, total cholesterol; TG, triglyceride; LDL-C, low-density lipoprotein cholesterol; HDL-C, high-density lipoprotein cholesterol; AST, aspartate aminotransferase; ALT, alanine aminotransferase.

### Correlations between serum NGAL levels and clinical and laboratory parameters

Correlations between the maternal serum NGAL levels and clinical and laboratory parameters in the GDM group and non-GDM group are presented in **Table 2**. Pearson correlation analysis showed that serum NGAL levels were positively correlated with pre-pregnancy BMI and HbA1c, fasting insulin, TG, TC, HOMA-IR, OGTT glucose and LDL-C levels in the GDM group (*P* < 0.05). However, there were no significant correlations between serum NGAL levels and other clinical and laboratory parameters in the GDM group (P > 0.05). In addition, no correlations were observed between maternal serum NGAL levels and clinical and laboratory parameters in the non-GDM group.

**Table 2:** Correlations between serum NGAL levels and clinical and laboratory parameters in the two groups.

Variable	GDM group (n=74)		non-GDM group (n=414)	
	*r*	*p-value*	*r*	*p-value*
Pre-pregnancy BMI (kg/m^2^)	0.411	**<0.001**	0.083	0.532
SBP (mm/Hg)	-0.131	0.418	-0.247	0.342
DBP (mm/Hg)	0.081	0.336	0.135	0.415
HbA1c (%)	0.341	**0.012**	0.179	0.132
Fasting insulin (mIU/ml)	0.672	**<0.001**	0.154	0.253
Fasting glucose (mmol/L)	0.713	**0.021**	0.214	0.314
1 h-glucose (mmol/L)	0.801	**0.032**	0.143	0.146
2 h-glucose (mmol/L)	0.632	**0.019**	0.227	0.273
HOMA-IR	0.631	**<0.001**	-0.337	0.371
TC (mmol/L)	0.268	**0.032**	0.153	0.214
TG (mmol/L)	0.391	**<0.001**	0.132	0.115
LDL-C (mmol/L)	0.513	**0.017**	0.112	0.421
HDL-C (mmol/L)	-0.132	0.552	-0.274	0.218
AST (U/L)	0.222	0.118	0.172	0.098
ALT (U/L)	0.371	0.243	0.762	0.327
Creatinine (μmol/L)	0.432	0.347	0.271	0.514
Uric acid (μmol/L)	0.586	0.175	0.192	0.205

P values were calculated by Pearson correlation analysis. Statistically significant values at P < 0.05 are shown in bold.

BMI, body mass index; SBP, systolic blood pressure; DBP, diastolic blood pressure; HbA1c, glycated haemoglobin; HOMA-IR, homeostasis model assessment-insulin resistance; TC, total cholesterol; TG, triglyceride; LDL-C, low-density lipoprotein cholesterol; HDL-C, high-density lipoprotein cholesterol; AST, aspartate aminotransferase; ALT, alanine aminotransferase.

### Clinical and laboratory risk factors for GDM explored by univariable logistic regression

Univariable logistic regression was carried out to identify the risk factors for GDM. The results showed that maternal age and pre-pregnancy BMI were significantly and positively correlated with the risk of GDM, with ORs of 3.142 (95% CI = 0.812-7.143) and 4.318 (95% CI = 0.914-8.317), respectively. Regarding laboratory parameters, HbA1c, HOMA-IR, TG, and LDL-C levels were significantly and positively correlated with the risk of GDM (P<0.05) (**Table 3**). These findings suggested that pre-pregnancy BMI, maternal age, and HbA1c, HOMA-IR, TG, and LDL-C levels are risk factors for GDM.

**Table 3:** Clinical and laboratory risk factors for GDM explored by univariable logistic regression.

Parameters	OR	95% CI	*p-value*
Maternal age (years)	3.142	0.812-7.143	**0.013**
Gestational age (weeks)	3.271	0.337-10.165	0.428
Pre-pregnancy BMI (kg/m^2^)	4.318	0.914-8.317	**<0.001**
SBP (mm/Hg)	1.318	0.227-7.025	0.332
DBP (mm/Hg)	2.102	0.153-9.067	0.298
Parity			
Primiparous	1.000	Reference	–
Multiparous	1.287	0.318-8.132	0.465
HbA1c (%)	2.292	0.104-5.145	**0.002**
Fasting insulin (mIU/ml)	3.321	0.227-9.172	0.312
HOMA-IR	4.732	0.106-10.287	**<0.001**
TC (mmol/L)	7.293	1.007-18.023	0.127
TG (mmol/L)	3.784	0.803-10.174	**0.002**
LDL-C (mmol/L)	4.313	0.514-12.637	**0.007**
HDL-C (mmol/L)	1.145	0.125-7.472	0.226
AST (U/L)	2.773	0.104-9.381	0.114
ALT (U/L)	1.467	0.187-8.451	0.179
Creatinine (μmol/L)	7.578	1.241-15.752	0.225
Uric acid (μmol/L)	4.331	0.453-10.713	0.341

P values were calculated by univariable logistic regression analysis after adjustment for potential confounding factors, including a family history of diabetes, dietary habits, physical activity during pregnancy, and economic status. Statistically significant values at P < 0.05 are shown in bold.

BMI, body mass index; SBP, systolic blood pressure; DBP, diastolic blood pressure; HbA1c, glycated haemoglobin; HOMA-IR, homeostasis model assessment-insulin resistance; TC, total cholesterol; TG, triglyceride; LDL-C, low-density lipoprotein cholesterol; HDL-C, high-density lipoprotein cholesterol; AST, aspartate aminotransferase; ALT, alanine aminotransferase.

**Figure 2:**
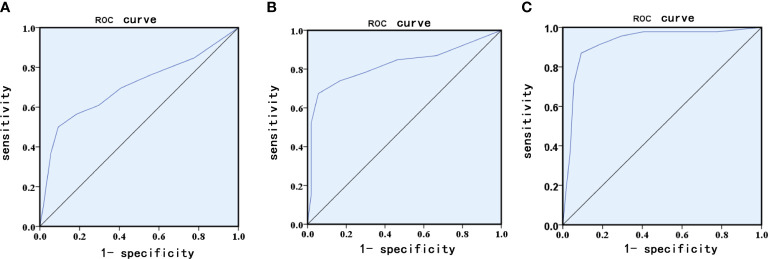
Receiver operating characteristic (ROC) curves for logistic regression models utilizing clinical and laboratory risk factors **(A)**, serum NGAL **(B)** serum NGAL levels and clinical and laboratory risk factors **(C)**.

According to univariable logistic regression, we used these identified risk factors to build a risk prediction model for GDM. An ROC curve was constructed using the model with an AUC of 0.705 (sensitivity of 55.3% and specificity of 91.3%, 95% CI=0.598–0.812, P<0.001, **Figure 2A**).

### Association of serum NGAL levels with the risk of GDM

The association of serum NGAL levels with the risk of GDM was assessed by a logistic regression model. Serum NGAL levels were divided into tertiles according to the cut-off points of the distribution of serum NGAL levels. The lowest tertile was considered as a reference.

**Figure 3:**
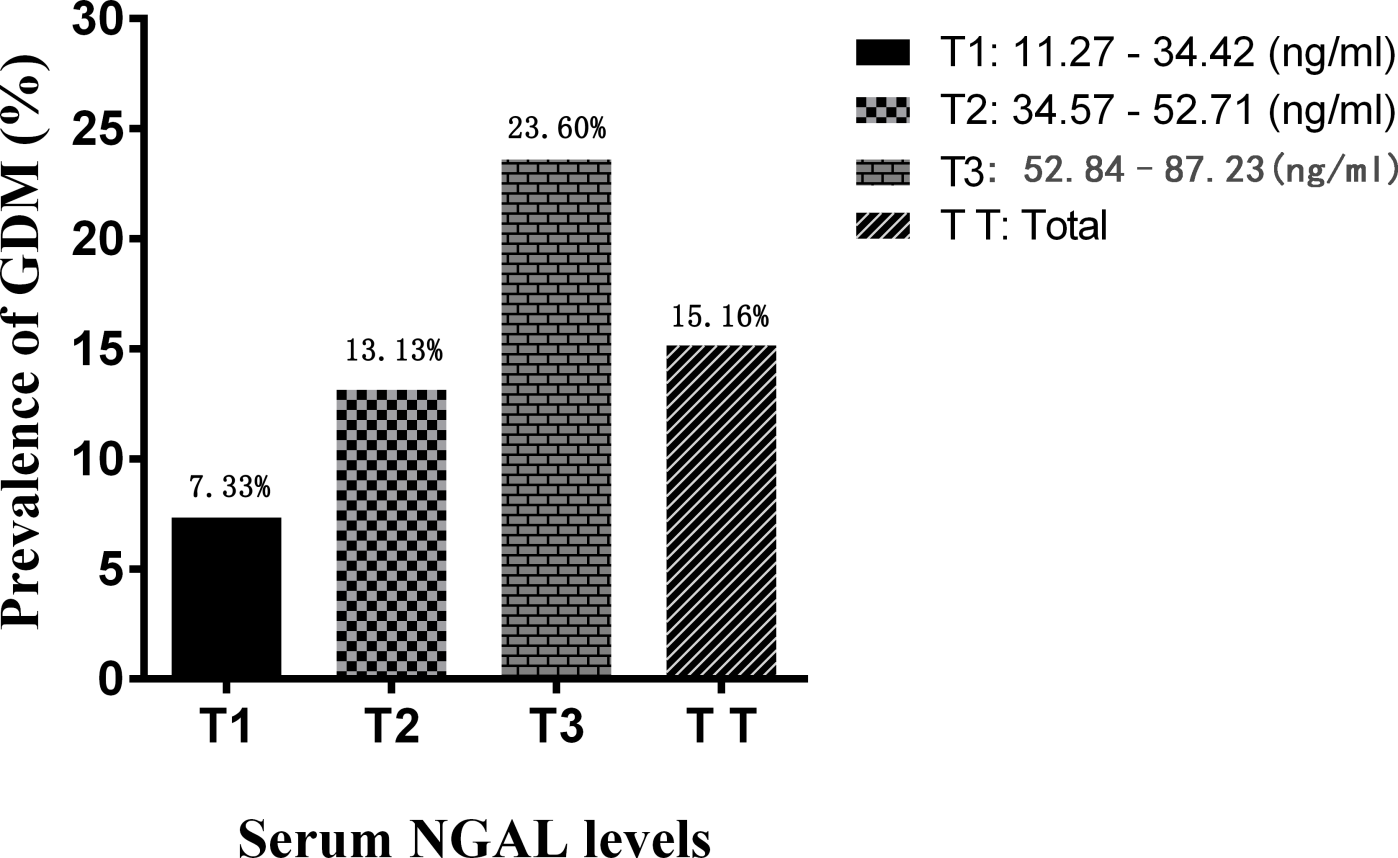
Prevalence of GDM for each tertile of serum NGAL levels.

The results showed that the prevalence of GDM increased stepwise from 7.33% to 23.60% across serum NGAL tertiles, with a threefold increase in the highest tertile versus the lowest tertile (**Figure 3**). Women with serum NGAL levels in the highest tertile as well as those with levels in the middle tertile had a higher risk of GDM than those with levels in the lowest tertile, with adjusted ORs of 4.513 (95% CI = 1.209-10.224) and 2.231 (95% CI = 0.814-6.217), respectively (**Table 4**). When regarded as a continuous variable, serum NGAL levels were significantly associated with an increased risk of GDM (adjusted OR = 1.512, 95% CI = 0.491–3.013, *P* = 0.012).

**Table 4:** Association of serum NGAL levels with the risk of GDM.

Serum NGAL levels (ng/ml)	GDM, n (%)	adjusted OR	95% CI	*p-value*
T1: 11.27 - 34.42	11 (7.33)	1	Reference	**-**
T2: 34.57 - 52.71	21 (13.13)	2.231	0.814-6.217	**0.017**
T3: 52.84 – 87.23	42 (23.60)	4.513	1.209-10.224	**<0.001**
As a continuous variable	–	1.512	0.491–3.013	**0.012**

P values were calculated by logistic regression analysis after adjustment for potential confounding factors, including pre-pregnancy BMI, maternal age, and HbA1c, HOMA-IR, TG, and LDL-C levels. Statistically significant values at P < 0.05 are shown in bold.

### Association of serum NGAL levels with the risk of adverse pregnancy outcomes

Previous studies have reported that GDM is associated with a series of adverse pregnancy outcomes, such as foetal growth restriction, large for gestational age, caesarean section, preterm delivery, postpartum haemorrhage, polyhydramnios, preeclampsia, and preterm premature rupture of membranes. Thus, we also investigated the associations between serum NGAL levels and these adverse pregnancy outcomes. The results showed that compared to the lowest tertile, the middle and highest tertiles of serum NGAL levels were associated with a higher risk of these adverse pregnancy outcomes, except foetal growth restriction and preeclampsia (**Table 5**).

**Table 5:** Association of serum NGAL levels with the risk of adverse pregnancy outcomes.

Adverse pregnancy outcomes	Serum NGAL levels (ng/ml)			
	T1 (11.27 - 34.42)OR (95% CI)	T2 (34.57 - 52.71)OR (95% CI)	T3 (52.84 -87.23)OR (95% CI)	*p-value*
FGR	1.00 (reference)	0.94 (0.23–6.27)	1.58 (0.12–6.35)	0.341
LGA	1.00 (reference)	2.51 (0.21–4.36)	4.71 (1.313–8.47)	**0.001**
CS	1.00 (reference)	2.73 (1.05–7.42)	3.21 (1.14–9.04)	**0.028**
PD	1.00 (reference)	1.78 (0.18–5.16)	2.81 (0.26–6.13)	**0.012**
PH	1.00 (reference)	2.13 (0.31–6.13)	3.17 (1.04–6.52)	**0.021**
PHD	1.00 (reference)	2.34(0.37–5.21)	4.51 (1.31–7.04)	**0.001**
PE	1.00 (reference)	0.67 (0.15–5.12)	1.04 (0.21–4.15)	0.122
PPROM	1.00 (reference)	2.71 (0.31–6.15)	4.05 (1.14–8.19)	**0.003**

P values were calculated by logistic regression analysis after adjustment for potential confounding factors, including gravidity, parity, gestational age, family history of diabetes, pre-pregnancy BMI, and TC, TG, HbA1c, HDL-C, and LDL-C levels.

OR, odds ratio; CI, confidence interval; FGR, foetal growth restriction; LGA, large for gestational age; CS, caesarean section; PD, preterm delivery; PH, postpartum haemorrhage; PHD, polyhydramnios; PE, preeclampsia; PPROM, preterm premature rupture of membranes.

### Predictive model construction and evaluation of the prediction efficacy of serum NGAL levels for GDM

To evaluate the prediction efficacy of serum NGAL levels for GDM, ROC curves were constructed using the serum NGAL level model with an AUC of 0.823 (sensitivity of 96.3% and specificity of 82.2%, 95% CI=0.733–0.914, P<0.001, **Figure 2B**). Therefore, the performance of the model constructed by using serum NGAL levels was greater than that of the model constructed by using the identified risk factors, including prepregnancy BMI, maternal age, and HbA1c, HOMA-IR, TG, and LDL-C levels (**Figure 2A**). When the serum NGAL level was combined with the identified risk factors, the combined risk prediction model achieved an AUC of 0.923 (sensitivity of 91.2% and specificity of 90.8%, 95% CI=0.863-0.982, P<0.001, **Figure 2C**).

## Discussion

Our study found that serum NGAL levels in the first trimester were significantly higher in women who later developed GDM than in those who did not develop GDM. Moreover, a positive association was found between serum NGAL levels in the first trimester and the risk of GDM after adjustment for potential confounding factors. We also used serum NGAL levels to construct a risk prediction model for GDM, and the model achieved excellent performance, with an AUC of 0.823. These findings indicate that serum NGAL in the first trimester of pregnancy is a potential new biomarker for the prediction of GDM.

NGAL is an adipocytokine that is highly expressed in adipose tissues and implicated in various metabolic and inflammatory diseases (9, 29). Previous studies reported that serum NGAL levels were significantly elevated in type 2 diabetes mellitus patients and that NGAL levels exhibited a positive correlation with obesity, hypertriglyceridaemia, hyperglycaemia, and insulin resistance (16, 30). Xiaoqian Yin et al. reported that maternal serum NGAL levels were significantly higher in women with GDM than in those without GDM and correlated positively with fasting plasma glucose levels in the third trimester (24). Lou et al. demonstrated that plasma NGAL levels were significantly increased in women with GDM, particularly among those with a pre-pregnancy BMI over 25 kg/m^2^ (25). Our results were consistent with these studies since we found that serum NGAL levels and insulin resistance index scores in the first trimester were significantly higher in women who later developed GDM than in those who did not develop GDM. Moreover, we also found that serum NGAL levels were positively correlated with pre-pregnancy BMI and HbA1c, fasting insulin, TG, TC, HOMA-IR, OGTT glucose and LDL-C levels in the first trimester in women who later developed GDM. These findings suggest that NGAL may be an indicator of disorders of glucose metabolism, lipid metabolism, and insulin resistance, which are closely associated with GDM (31–33).

Although the study has revealed that serum NGAL levels are positively correlated with glucose metabolism disorders in women with GDM, the main mechanism for mediating NGAL expression is largely unknown. Zhang et al. studied the metabolic regulation of NGAL production in adipocytes and found that insulin can promote the expression and secretion of NGAL in a dose-dependent manner (34). Similarly, another study by Tan et al. showed that insulin treatment in a conditioned medium could significantly increase the secretion of NGAL protein in omental adipose tissue *in vitro* in a dose-dependent manner (35). Furthermore, they found that circulating NGAL levels were also increased in human subjects after insulin treatment, and the mechanisms involved in insulin-induced NGAL expression were the activation of phosphoinositide 3-kinase (PI3K) and mitogen-activated protein kinase (MAPK) signalling pathways (35). However, other studies demonstrated that insulin induces NGAL expression by promoting glucose metabolism (oxidation) and the production of reactive oxygen species (ROS) and then activating the NF-κB signalling pathway (34). This has also been confirmed by other studies. Studies by Zhao et al. provided direct evidence that NF-κB bonded to the NGAL promoter of human adipocytes and that the NF-κB signalling pathway was activated when insulin induced NGAL expression (15, 36).

Insulin resistance occurs when normal concentrations of insulin fail to achieve an appropriate biological response downstream of the insulin receptor. As a result, β-cells must release more insulin than usual to modulate glucose homeostasis, ultimately leading to hyperinsulinaemia (37). In fact, the presence of insulin resistance is observed long before the clinical manifestations of GDM (38). Therefore, in pregnant women who later develop GDM, insulin resistance and hyperinsulinaemia may already be present in the first trimester. Our study confirmed that serum insulin levels and insulin resistance assessed by HOMA-IR in the first trimester were significantly increased in women who later developed GDM compared with those who did not develop GDM. These results suggest that insulin resistance and hyperinsulinaemia may be responsible for the elevated levels of NGAL in the first trimester of women who later develop GDM. This finding indicates that serum NGAL in the first trimester of pregnancy is a potential new biomarker for the prediction of GDM.

Over the years, numerous biomarkers, such as metabolic biomarkers, inflammatory biomarkers, and placental biomarkers, have been identified for the prediction of GDM (39–41). However, none of these biomarkers have sufficient validity for clinical practice (7). In this study, we found that serum NGAL in the first trimester of pregnancy is a potential new biomarker for the prediction of GDM, and the prediction model achieved great performance. We hope that this can be further verified in clinical trials in future studies and ultimately help guide the clinical practice of antenatal care.

Our study has many strengths. First, our study was conducted using a prospective design, which could avoid the recall bias that is usually present in a retrospective study. Second, we focused on the early prediction of GDM in the first trimester before OGTT screening. Thus, this study has clinical practical value and can provide a reliable basis for early clinical decision-making. Third, all laboratory and clinical measurements were carried out according to standardized procedures with high reliability. Fourth, our study constructed a risk prediction model for predicting GDM before OGTT screening by evaluating serum NGAL levels in the first trimester; the model achieved excellent performance. Nevertheless, our study also has limitations. First, although we adjusted for many potential confounding factors, we cannot rule out the possible influence of other unmeasured factors on the results. Second, we did not investigate the underlying mechanisms that mediate the association between serum NGAL levels and the risk of GDM. Finally, since our participants were mainly of Han ethnicity, our findings may not be generalizable to other racial groups when considering different dietary habits among racial groups.

## Conclusions

Our findings demonstrated a positive association between serum NGAL levels in the first trimester of pregnancy and later GDM risk. We further used the observed association to construct a risk prediction model for GDM, which achieved excellent performance. This study suggests that maternal serum NGAL in the first trimester of pregnancy can serve as an early predictive biomarker for GDM, which could help guide the clinical practice of antenatal care.

## Data availability statement

The original contributions presented in the study are included in the article/Supplementary Material. Further inquiries can be directed to the corresponding authors.

## Ethics statement

The studies involving human participants were reviewed and approved by Ethics Committee of The First Affiliated Hospital of Chengdu Medical College (No. CYFY17032024). The patients/participants provided their written informed consent to participate in this study.

## Author contributions

LL contributed to the study design and interpretation of the data. CL and JD contributed to the collection of data. JL and CH contributed to the drafting and revision of the manuscript. All authors contributed to the article and approved the submitted version.

## Funding

This work was supported by grants from The First Affiliated Hospital of Chengdu Medical College (No. CYFY-GQ24, CYZYB20-17), Sichuan Medical Committee (No. Q20075), and Chengdu Science and Technology Bureau (No. 2021-YF05-00269-SN).

## Conflict of interest

The authors declare that the research was conducted in the absence of any commercial or financial relationships that could be construed as a potential conflict of interest.

## Publisher’s note

All claims expressed in this article are solely those of the authors and do not necessarily represent those of their affiliated organizations, or those of the publisher, the editors and the reviewers. Any product that may be evaluated in this article, or claim that may be made by its manufacturer, is not guaranteed or endorsed by the publisher.
